# Molecular Mechanisms and Current Treatment Options for Cancer Cachexia

**DOI:** 10.3390/cancers14092107

**Published:** 2022-04-23

**Authors:** Syed Sayeed Ahmad, Khurshid Ahmad, Sibhghatulla Shaikh, Hye Jin You, Eun-Young Lee, Shahid Ali, Eun Ju Lee, Inho Choi

**Affiliations:** 1 Department of Medical Biotechnology, Yeungnam University, Gyeongsan 38541, Gyeongsangbuk-do, Korea; sayeedahmad4@gmail.com (S.S.A.); ahmadkhursheed2008@gmail.com (K.A.); sibhghat.88@gmail.com (S.S.); 2 Research Institute of Cell Culture, Yeungnam University, Gyeongsan 38541, Gyeongsangbuk-do, Korea; ali.ali.md111@gmail.com; 3 Tumor Microenvironment Branch, Division of Cancer Biology, Research Institute, National Cancer Center, 323 Ilsan-ro, Ilsandong-gu, Goyang 10408, Gyeonggi-do, Korea; hjyou@ncc.re.kr (H.J.Y.); 74673@ncc.re.kr (E.-Y.L.); 4 Department of Cancer Biomedical Science, Graduate School of Cancer Science and Policy, National Cancer Center, 323, Ilsan-ro, Ilsandong-gu, Goyaan 10408, Gyeonggi-do, Korea

**Keywords:** cancer cachexia, skeletal muscle, inhibitors, myostatin, natural compounds

## Abstract

**Simple Summary:**

The primary characteristics of cancer cachexia are weakness, weight loss, atrophy, fat reduction, and systemic inflammation. Cachexia is strongly associated with cancers involving the lungs, pancreas, esophagus, stomach, and liver, which account for half of all cancer deaths. TGF-β, MSTN, activin, IGF-1/PI3K/AKT, and JAK-STAT signaling pathways are known to underlie muscle atrophy and cachexia. Anamorelin (appetite stimulation), megestrol acetate, eicosapentaenoic acid, phytocannabinoids, targeting MSTN/activin, and molecules targeting proinflammatory cytokines, such as TNF-α and IL-6, are being tested as treatment options for cancer cachexia.

**Abstract:**

Cancer cachexia is a condition marked by functional, metabolic, and immunological dysfunctions associated with skeletal muscle (SM) atrophy, adipose tissue loss, fat reduction, systemic inflammation, and anorexia. Generally, the condition is caused by a variety of mediators produced by cancer cells and cells in tumor microenvironments. Myostatin and activin signaling, IGF-1/PI3K/AKT signaling, and JAK-STAT signaling are known to play roles in cachexia, and thus, these pathways are considered potential therapeutic targets. This review discusses the current state of knowledge of the molecular mechanisms underlying cachexia and the available therapeutic options and was undertaken to increase understanding of the various factors/pathways/mediators involved and to identify potential treatment options.

## 1. Introduction

The primary characteristics of cancer cachexia (CC), which accounts for ~22% of cancer deaths, are weakness, weight loss, atrophy, fat reduction, and systemic inflammation [1,2]. Cachexia is strongly associated with cancers involving the lungs, pancreas, esophagus, stomach, and liver, which account for half of all cancer deaths. Furthermore, several malignancy-associated conditions, such as chronic obstructive pulmonary disease (COPD), chronic infections (AIDS and tuberculosis), heart failure, and rheumatoid arthritis, cause inflammation, anorexia, hypogonadism, and other symptoms, all of which lead to muscle wasting and fat loss [3,4]. Earlier, we investigated the potential use of SM mass loss as a marker of several diseases, including diabetes, obesity, and aging [5,6,7]. Furthermore, it has been well established that multiple mediators generated by cancer cells are responsible for cachexia [8]. Prostaglandin E2 and pro-inflammatory cytokines such as interleukins (IL-1, IL-6), tumor necrosis factor (TNF), interferon, TNF receptor-associated factor 6, and other tumor-derived catabolic factors like activin and myostatin (MSTN) are examples of such mediators [8,9]. CC and starvation cause weight loss in different ways. Weight loss in cancer patients is due to approximately equal losses of adipose tissue and SM mass, whereas anorexia nervosa-associated weight loss is primarily due to fat loss (muscle loss is only a minor contributor) [10]. In addition, the incidence of CC is dependent on tumor type, for example, its prevalence in gastric/pancreatic, breast, and neck cancer are 80, 40, and 40%, respectively, and in lung, prostate, and colon cancer, its prevalence is around 50%. In some cases, leukemia patients also develop the syndrome. However, CC development is unrelated to tumor size [11,12,13,14].

By regulating glucose, protein, and fat metabolisms, insulin, insulin-like growth factor 1 (IGF-1), and growth hormones (GHs) have significant impacts on body composition. The signals generated by these molecules are disrupted in the presence of muscle wasting or cachexia, and this results in an anabolic/catabolic imbalance. In cachexia, insulin receptor, GH, and IGF-1 pathways; peroxisome proliferator-activated receptor gamma (PPARγ) agonists; angiotensin II inhibitors; and testosterone are possible therapeutic targets [15,16]. Transforming growth factor-beta (TGF-β), MSTN, activin, IGF-1/PI3K/AKT, and JAK-STAT signaling pathways are known to underlie muscle atrophy and cachexia [17]. Other potential mediators include testosterone and IGF-1 deficiency and excess MSTN and glucocorticoids [18]. To address the situation posed by limited treatment options, a deeper knowledge of the mechanism responsible for cachexia is required. At present, it appears that drug developments aimed at cachexia management should target anti-inflammatory and appetite-stimulating properties. We commence this review with a brief overview of CC and its characteristics. The molecular mechanisms underlying cachexia are discussed in detail, to improve understanding of the various factors implicated and identify possible therapeutic strategies.

## 2. Cancer Cachexia

**Figure 1:**
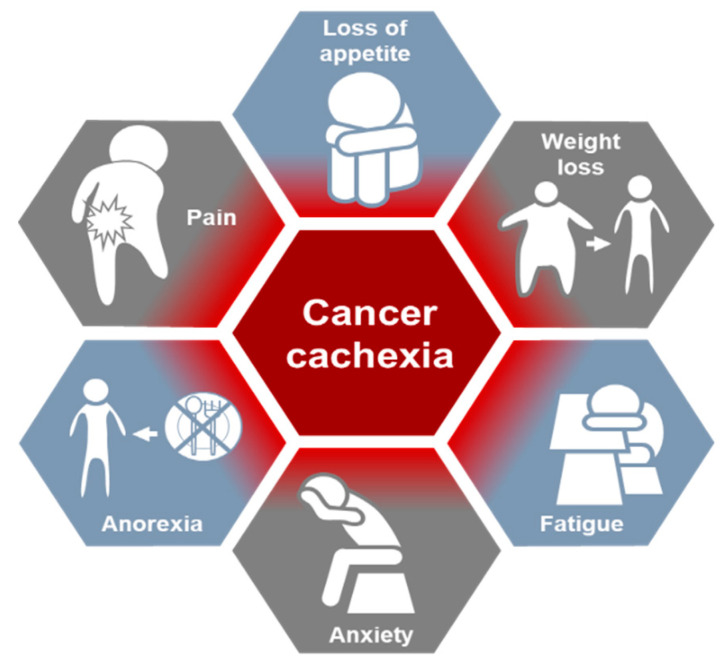
The common assessment for the clinical management of cancer cachexia.

Cachexia has been described as an imbalance between energy intake and expenditure leading to severe weight loss [3,8,19]. The condition is subdivided into three stages, precachexia, cachexia, and refractory cachexia. The diagnostic criteria of CC are (1) weight loss of >5% in 6 months in the absence of intended starvation, (2) a body-mass index (BMI) of <20 kg/m^2^ and progressive weight loss of >2%, and (3) a low SM index (sarcopenia) with continued weight loss of >2% during a measure of muscularity with fluid retention tumor mass and obesity. During precachexia, some signs like anorexia and impaired glucose tolerance resulting in unexpected weight loss are evident. Refractory cachexia is characterized by two features [19]: (1) a low-performance status, meaning that a patient is capable of only minimal self-care, confined to a bed or chair for >50% of waking hours, or is completely disabled and incapable of self-care [20]; (2) short life expectancy (less than 3 months) [19]. The key features of cachexia are anorexia, catabolic drivers resulting in muscle wasting, muscle mass and strength loss, and functional and psychosocial effects of cachexia [19,21]. Cachexia was mainly described by clinical experience and/or research vantage points, including weight, skeletal muscle, physical function, food intake, metabolism, inflammation, treatment intensity, quality of life, healthcare utilization, and survival [22]. The symptoms of CC are illustrated in Figure 1.

## 3. The Molecular Mechanism Underlying Cancer Cachexia

### 3.1. Crosstalk between IGF-1 and MSTN Signaling Pathways in Cancer Cachexia

**Figure 2:**
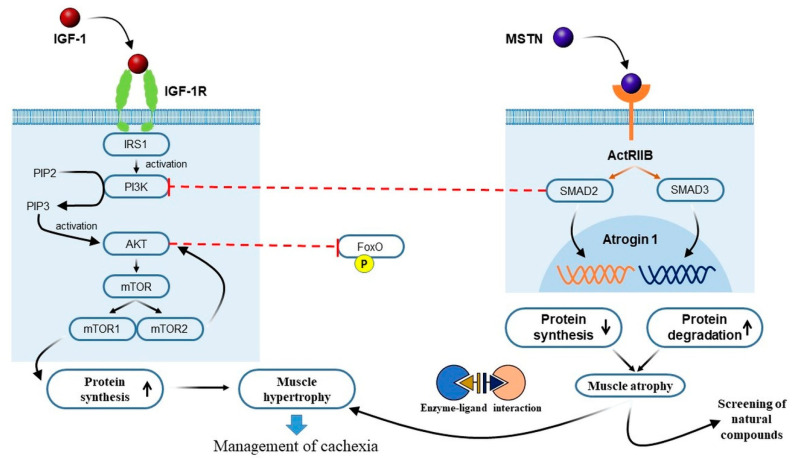
Molecular mechanisms regulated by IGF-1 and MSTN: Active Akt produces the mTOR signal, which leads to protein synthesis and inhibits (phosphorylates) FoxO. IGF-1 is primarily responsible for protein synthesis and muscle hypertrophy, whereas MSTN is responsible for protein degradation causing muscle atrophy. We suggest that screening of natural compounds and their derivatives for anti-MSTN activity might shift the balance toward muscle hypertrophy in cachexia.

Anabolic and catabolic pathways are regulated by IGF-1, a positive regulator of muscle growth [23]. Under normal circumstances, IGF-1 signaling dominates MSTN signaling, whereas MSTN overexpression inhibits IGF-1 [24,25,26]. IGF-1 stimulates protein synthesis in SM via the PI3K/Akt/mTOR and PI3K/Akt/GSK3 pathways, and the PI3K/Akt pathway inhibits FoxOs and suppresses the transcriptions of E3 ubiquitin ligases, which elicit protein breakdown via the ubiquitin-proteasome system. IGF-1 is also considered to suppress autophagy via a mammalian target of rapamycin (mTOR) and FoxO signaling [27]. Akt is involved in a variety of intracellular metabolic activities, which include hypertrophic responses to insulin and IGF-1. Furthermore, Akt has been identified as a crossing point between the MSTN and IGF-1 pathways [23,28,29]. In cachexia, IGF-1 signaling is impaired, because cachexic muscle cells do not respond to basic IGF-1 stimulation. Two strategies appear to be therapeutic candidates: (1) the utilization of PPAR-agonists to target post-receptor pathways or (2) the exploitation of alternate routes in muscle cells to access the same intracellular targets [15]. A schematic of the IGF1-Akt pathway is shown in Figure 2.

When IGF1 binds to its receptor, its intrinsic tyrosine kinase is activated and autophosphorylated, which results in the formation of insulin receptor substrate binding sites (IRSs). Phosphorylated IRSs aid the recruitment and activation of phosphatidylinositol-3-kinase (PI3K), which phosphorylates membrane phospholipids and converts phosphoinositide-4, 5-biphosphate (PIP3, which aids the activation of Akt) to PIP2. Thus, Akt stimulates protein synthesis via mTOR, which promotes protein synthesis and muscle hypertrophy [28].

In cases of chronic heart failure, circulating and local levels of MSTN, which play key roles in myocardial cachexia, are elevated [29]. Activin type-2 receptor (ActRIIB) antagonism and/or MSTN antibodies have emerged as viable therapeutic targets for the treatment of cachexia, although the broad clinical applications of these potential treatment strategies have not been demonstrated [30]. Furthermore, in mouse models, inhibiting ActRIIB reverses cachexia and improves survival [31]. MSTN has been introduced as a major interest in cachexia, sarcopenia, and muscle wasting conditions [32]. MSTN is released primarily by SM and, as mentioned above, negatively regulates muscle mass [33], as demonstrated by the development of cachexia in rodents’ systemically administered MSTN [27,34]. MSTN signaling is facilitated by ActRIIB and leads to the phosphorylations of SMAD2 and 3 [35,36]. Congestive heart failure is frequently accompanied by cardiac cachexia, and in rodent models and clinical trials, blocking MSTN appears to improve muscle size and strength significantly [37]. Thus, stimulating IGF-1 and blockading MSTN using natural compounds would promote muscle hypertrophy and provide a possible means of managing cachexia. As a result of these findings, MSTN has emerged as an important developmental target for the treatment of cachexia and muscle wasting disorders. Randomized controlled studies on MSTN antagonists are required [38,39]. By reducing AKT phosphorylation and so boosting the levels of active FoxO1, MSTN signaling reversed the IGF-1/PI3K/AKT hypertrophy pathway, allowing for increased expression of atrophy-related genes. The known atrophy-related genes are Atrogin1 and Glb1 [33]. MSTN expression is increased in the muscle of tumor-induced cachexia [31]. MSTN’s role in the development of CC has been little studied in humans, and it is now being studied clinically. A better knowledge of the etiology and heterogeneity of CC might lead to the development of intervention measures to prevent or treat this life-threatening illness.

### 3.2. The PI3K/Akt/mTOR Pathway and Cancer Cachexia

SM and cardiac muscle atrophy are considered hallmarks of CC [40], and a variety of agents have been reported to reduce muscle atrophy. In 2001, Bodine et al. reported that a phosphoinositide 3-kinase (PI3K)–protein kinase B (PKB, AKT)–mTOR cascade importantly regulated SM hypertrophy in vivo via the modulations of p70S6K and PHAS-1/4E-BP1 [41]. In CC patients that exhibited weight loss before surgery, PI3K/AKT signaling was diminished and protein synthesis in SM was reduced [42]. The signaling involved was elucidated based on improved understanding of the activities of mTOR [27,43,44], which is a downstream kinase in the IGF-1/PI3K/AKT pathway that acts as a hub for muscle regulation by coordinating the ubiquitin proteasome system and autophagy [45]. mTOR forms mTORC1 and mTORC2 complexes containing RAPTOR and RICTOR, respectively [43,44]. mTORC1 is responsible for protein biosynthesis by 4E-BPs and p70^S6K^ in growing cells and suppresses catabolic autophagy by regulating unc-51-like autophagy-activating kinase 1 (ULK1) and ATG13. Under conditions of nutrient deprivation, mTORC1 is inactivated, which leads to coordinated autophagosome initiation and subsequent lysosomal biogenesis [46].

IGF-1 stimulates the productions of SM proteins via PI3K/Akt/mTOR and PI3K/Akt/GSK3 pathways [27]. A ketogenic diet targets glucose metabolism in cancer cells, inhibits the IGF-1 and PI3K/AKT/mTOR pathways, and suppresses CC, muscular wasting, and tiredness [47]. Growth factors and nutrients activate AKT via PI3K-dependent mechanisms, which, in turn, activate mTOR and enhance muscle cell proliferation and protein synthesis under physiologic conditions. AKT (a serine/threonine kinase) plays a crucial role in myogenic differentiation, and in CC, the phosphorylations of mTOR and its substrates, S6 ribosomal protein, and 4EBP were reduced, irrespective of AKT activation [48,49,50,51]. Furthermore, these alterations in mTOR-related protein signaling pathways were followed by small increases in the protein levels of Beclin1, which is associated with autophagy. In addition, the mTOR signaling system has been shown to regulate myofiber production and development during muscle regeneration through kinase independent and dependent pathways, respectively [52,53].

### 3.3. Roles of Peroxisome Proliferator-Activated Receptors in Cancer Cachexia

Peroxisome proliferator-activated receptors (PPARs) are well-known transcription factors that belong to the nuclear receptor superfamily and have three isoforms, namely, α, δ, and γ [54,55]. PPARs regulate the transcriptions of a wide range of genes involved in inflammation, metabolism, proliferation, and the differentiation of various cells [56,57], and they are associated with a number of pathologies, including cancer, type 2 diabetes, atherosclerosis, and Alzheimer’s disease. Moreover, PPARs are frequently co-expressed to varying degrees in many tissues, including SM and adipose tissue [58,59,60].

PPARα is expressed in the liver, SM, heart, adipose tissue, kidney, and other tissues, and it plays crucial roles in fatty acid catabolism, glucose metabolism, and the regulation of energy consumption and inflammation. PPARα agonists, notably fibrates (e.g., clofibrate, fenofibrate, ciprofibrate, bezafibrate), are used to improve lipid metabolism and insulin sensitivity in metabolic syndrome [61]. Fenofibrate, a selective PPARα activator used to treat dyslipidemia in humans, has been shown to reduce inflammation in rheumatoid arthritis patients [62]. It prevents the development of CC in mice [63]. Fenofibrate treatment restored muscle mass and body weight loss in a non-small cell lung cancer mouse model exhibiting muscle wasting and mimicking human CC [63]. PPARβ/δ is expressed at varying levels in several tissues, most notably in SM, but is also expressed in the heart, skin, and gut, and it has a wider range of functions than PPARα [58]. PPARβ plays a critical regulatory role in intermediate metabolic processes and is also involved in differentiation, apoptosis, inflammation, and other cancer-related processes [64]. PPARβ agonists (e.g., GW501516) activate PPARβ and provide functional improvements in Duchenne muscular dystrophy (DMD) patients by increasing utrophin A (an autosomal homolog of dystrophin) expression [65]. DMD is a serious, progressive muscle-wasting ailment that causes movement problems and premature death. Mutations in DMD (encoding dystrophin) cause the ailment by preventing dystrophin synthesis in the muscle. Muscles lacking dystrophin are more vulnerable to injury, resulting in a gradual loss of muscle structure and function [66,67].

PPARγ is expressed in the SM, placenta, lung, spleen, heart, liver, ovary, and other tissues but is most abundant in adipose tissue. PPARγ regulates whole-body glucose homeostasis and insulin sensitivity, and currently, studies have focused on its involvements in inflammation, lipid metabolism, and tumor development, particularly in the context of CC. [68,69]. PPARγ activation has an anti-inflammatory effect caused by attenuation of the NF-κB signaling pathway, and thus, it inhibits the productions of IL-6 and other pro-inflammatory factors by regulating the STAT3 pathway [70]. It was recently reported that alpinetin (a plant-derived flavonoid) retards CC progression and protects against muscle atrophy by activating PPARγ, thus suppressing the phosphorylations of NF-κB and STAT3 [71], and alantolactone was found to inhibit the STAT3 pathway to improve the muscular atrophy in a CC [72]. STAT3 is a transcription factor that promotes cancer growth and muscular cachexia. The deletion of the gene producing the STAT3 protein lowers the expression of muscle differentiation factors like MyoD and myogenin in vitro. In vivo investigations show that STAT3 deletion impairs post-traumatic muscle regeneration, which is consistent with previous findings [73]. STAT3 is the most important component of the IL-6 and JAK2 signaling pathways, controlling SM mass, growth, repair, and regeneration [74]. The STAT3 pathway has been demonstrated to cause muscle atrophy in DMD, Merosin-negative congenital muscular dystrophy (MDC1A), sepsis, and cancers [75]. The key molecular mechanism leading to CC is thought to be permanent stimulation of the acute phase protein response. In experimental cachexia models, the IL-6/STAT3 6 signaling pathway causes muscle mass loss [76]. A variety of STAT3-interacting peptides, such as PY*LKTK [77] and Y*LPQTV [78], are now being explored in preclinical trials. These peptides bind to the SH2 domain and hence inhibit STAT3 dimerization. Another STAT3 inhibitor, galiellalactone [79], binds to the DNA-binding domain responsible for STAT3 binding to DNA, preventing transcriptional activation of STAT3-targeted genes [80]. STAT3 activity is required for muscle tissue formation and maintains homeostasis, whereas STAT3 inhibitors appear to be potential components in illnesses involving muscle atrophy.

PPARγ activation induces preadipocyte to adipocyte differentiation and promotes triglyceride accumulation. Furthermore, PPARγ is an important transcription factor, and its inactivation explains the downregulations of multiple adipogenic genes. The expression and role of MALAT1 (metastasis-associated lung adenocarcinoma transcript 1) in adipocytes was recently studied by a microarray analysis, and MALAT1 knockdown was found to reduce adipogenesis by regulating PPARγ gene expression at the transcriptional level [81]. MALAT1 is a widely expressed lncRNA, and it participates in a variety of physiological and pathological processes, such as myogenesis, cancer, and aortic aneurysm [82]. Like some well-known proteins, such as hormone-sensitive lipases, adipose triglyceride lipases, and uncoupling protein-1, and lncRNAs (e.g., CAAlnc1), they are promising unique regulators of adipose tissue loss in CC [83]. In one study, PPARγ expression was markedly enhanced in the SM of tumor-bearing mice, which demonstrated the significance of the effect of PPARγ on muscle wasting. In addition, the administration of GW1929 (a PPARγ agonist) resulted in the restoration of muscle loss [84]. Furthermore, PPARγ expression was found to be significantly elevated in mesenchymal glioblastoma, which suggested the potential use of PPARγ as a therapeutic target [69]. Taken together, the above-mentioned observations indicate that PPARγ, PPARα, and PPARβ/δ are potential therapeutic targets in cancer-associated cachexia.

## 4. Treatment Options for Cancer Cachexia

There is still no gold standard treatment for CC because of the variety of cancer types and pathophysiological processes involved, and thus, the condition is difficult to manage. A number of therapeutic options are available, but their benefits appear to be limited, and more clinical evidence is needed before their efficacies can be determined [85]. Here, we categorize treatment options for CC into groups based on their effects on appetite, inflammatory cytokines, and metabolism.

CC is treated using multimodal rather than single approaches because of its multifaceted nature [86]. These methods involve different treatment combinations, such as combinations of pharmaceuticals, nutritional supplements, specific diets, and modest physical activity, which appear to act synergistically and, in some cases, effectively restore metabolic alterations and alleviate anorexia [12,86,87,88]. However, defining beneficial combinations of medicines, nutrition, and exercise will require much research effort [88]. Many medicines have been suggested and subjected to clinical trials. These include appetite stimulants, steroids, thalidomide, cytokine inhibitors, branched chain amino acids, nonsteroidal anti-inflammatory agents, eicosapentaenoic acid, and anti-serotoninergic drugs [89]. These medicines are medroxyprogesterone (500 mg/day) or megestrol acetate (320 mg/day) and oral supplementation with eicosapentaenoic acid, L-carnitine (4 g/day), and thalidomide (200 mg/day) [90]. Table 1 provides a list of the immunomodulatory factors used to manage cachexia. Personalized physical exercise in conjunction with pharmacological and nutritional assistance has the potential to be beneficial. A better knowledge of the pathogenetic mechanisms that cause CC-related muscle wasting will allow for the development of a targeted mechanism-based multimodal strategy that can be used early and effectively.

### 4.1. Appetite Stimulation Using Anamorelin

Anamorelin is an orally prescribed ghrelin receptor agonist that is considered to ameliorate CC by increasing appetite [103]. Anamorelin hydrochloride, a ghrelin receptor agonist with a small molecular weight (583.2 g/mol), has shown excellent results in recent Phase III studies and has regulatory clearance in Japan for the treatment of cachexia [103]. It improves CC by raising serum IGF-1 and boosting appetite. Ghrelin is a 28 amino acid hormone and was first identified in a rat stomach extract as an endogenous ligand of GH secretagogue receptor 1a [104]. Ghrelin binding to this receptor leads to GH release, which is closely related to systemic metabolism [104,105]. As a nutrient sensor, ghrelin and its receptor axis influence a variety of metabolic processes [105]. In CC, ghrelin is considered a potential therapeutic option and has been investigated in many cancers, including colon and non-small cell lung cancer [106,107,108]. Pralmorelin (Kaken Pharma, Tokyo, Japan, and Sella Pharma, Schio, Italy) has been approved for GH deficiency, and macimorelin (Aeterna Zentaris, Charleston, SC, USA), anamorelin (Helsinn, Lugano, Switzerland), and relamorelin (Rhythm, Saitama, Japan) are undergoing preclinical or clinical trials [105].

### 4.2. Megestrol Acetate

Megestrol acetate is a synthetic progestin and appetite stimulant that was approved for the treatment of anorexia, cachexia, or weight loss due to unidentified causes in 1993 [109,110]. Recently, Ruiz-Garcia et al. reviewed megestrol acetate clinical trials, and according to their report, megestrol acetate resulted in weight gains but did not improve quality of life. In another study, megestrol acetate was also found induce weight gains, though it was mentioned that its dosage required optimization [110].

### 4.3. Eicosapentaenoic Acid (EPA) Supplementation

Eicosapentaenoic acid (EPA) is a polyunsaturated fatty acid found in some fish oils, and it has been suggested to promote body weight gain in CC. However, five trials conducted on the topic failed to demonstrate that EPA therapy results in weight gain in cachexia patients [111]. Nonetheless, the effect of EPA supplementation on appetite stimulation in CC is being investigated [112,113,114].

### 4.4. Systemic Inflammation

As mentioned above, loss of body weight and BMI reduction resulting from adipose tissue and SM loss are major symptoms of CC. Several cytokines, e.g., TNF-α, IL-1, and IL-6, secreted by immune or tumor cells of gastric, pancreatic, and other cancers have been shown to be associated with wasting of mesenchymal tissues [8,115]. TNF-α and IL-6 have a role in the development of SM atrophy and fat depletion in CC patients [72]. The metabolism of glucose, protein, and fat is altered in the CC state. TNF-α causes an increase in gluconeogenesis, adipose tissue loss, and proteolysis, as well as a reduction in protein, lipid, and glycogen synthesis [115]. General immune suppressors have also been considered as treatments for cachexia, and molecules targeting proinflammatory cytokines, such as TNF-α and IL-6, are being tested as treatment options.

### 4.5. TNF-α Inhibitors

TNF-α has been suggested to be involved in CC [116] muscle atrophy, the inhibition of adipocyte and skeletal myocyte differentiation, and insulin resistance, and thus, its targeting has been investigated as a possible therapeutic strategy for CC [8,117,118]. TNF-α signaling has been antagonized using etanercept (which targets the TNF-α receptor) and using infliximab, adalimumab, golimumab, or certolizumab-pegol, which target TNF-α [119]. These agents are currently undergoing preclinical or clinical trials singly or in combination with other drugs (e.g., gemcitabine) in non-small lung cancer, pancreatic cancer, and other cancers [120]. Nevertheless, despite accumulating positive evidence, this topic remains under investigation.

### 4.6. IL-6 Inhibitors

By promoting the differentiation of immune cells such as B and T cells and stimulating the productions of C-reactive protein, serum amyloid A, fibrinogen, and other proteins in hepatocytes, IL-6 is a major player in the acute immune response [121]. This cytokine has also been suggested to participate in the pathophysiologies of chronic inflammatory diseases. For example, IL-6 stimulates signaling for osteoclast activation and differentiation, angiogenesis by stimulating vascular endothelial growth factor production leading to vascular permeabilization in cancer and rheumatoid arthritis, and keratinocyte proliferation resulting in skin fibrosis [117,121,122]. Furthermore, elevated circulatory IL-6 levels have been reported to be associated with muscle atrophy caused by the suppression of muscle protein synthesis in mice, which suggests another option for the treatment of cachexia [85]. Humanized antibodies against IL-6 receptor (tocilizumab) or soluble IL-6 (sirukumab, olokizumab, or clazakizumab) are currently undergoing preclinical or clinical studies [85,118,120,123,124,125].

### 4.7. Phytocannabinoids

The genus *Cannabis* is classified into three main species, the fiber-type *C. sativa* L; the drug type *C. indica* Lam, which contains high levels of the psychoactive drug Δ-9-tetrahydrocannabinol (THC); and *C. ruderalis* Janisch [126,127]. Central nervous system and peripheral tissue studies have shown that interactions between cannabidiol or THC and their receptors, cannabidiol receptor 1 and 2 (CB1 and CB2; G-protein coupled receptors that signal through cyclic AMP (cAMP) and Ca^2+^ ions), also have anti-inflammatory, antioxidative, and immune response-ameliorating effects [126]. Both cannabidiol and THC have been investigated as treatments for cachexia, but their effects are not clear, and further systemic validation is required [118,124].

### 4.8. Thalidomide

Since the anti-angiogenic effects of thalidomide were demonstrated in vivo [128], thalidomide has been used as an anti-cancer drug [125]. In addition, thalidomide and its analogs (lenaliomide and pomalidomide) influence immunomodulation by regulating TNF-α, TGF-β, IL-1β, IL-6, granulocyte macrophage colony-stimulating factor, and T cell proliferation and function [129]. Thalidomide inhibits ubiquitin ligase (E3) and leads to uncontrolled protein degradation, which suggests applications in CC and cancer progression [130]. In cachexia, thalidomide has been shown to increase body weight and appetite. However, large randomized combinatorial drug trials are required in patients with advanced cancer [125,131].

### 4.9. Corticosteroids

According to the American Society of Clinical Oncology (ASCO) guidelines for the management of CC, targeting corticosteroids is recommended for patients with advanced cancer [125]. By modulating energy reserves through a variety of pathways, glucocorticoids are key players in the hypothalamic–pituitary–adrenal axis [85,132]. However, they also induce muscle atrophy by accelerating muscle protein degradation through autophagy and the ubiquitin–proteasome pathway and by attenuating protein synthesis in muscles [132,133,134]. Thus, further studies are required to determine the optimal doses of corticosteroids when administered in combinations with other drugs for cachexia and to validate their effectiveness.

### 4.10. Targeting Myostatin and Activin

Loss of SM mass is closely associated with weight loss in CC and non-responsiveness to increased appetite or nutritional supplementation [135,136,137,138]. The TGFβ family members MSTN and activin A have been most studied. MSTN (also known as growth differentiation factor-8) is a member of the TGFβ family, and it negatively regulates muscle differentiation by binding to its receptor, ActRIIB, which activates Smad2/3-linked signaling cascades, leading to muscle loss [8,139]. Activin A and MSTN interactions with ActRIIB have been studied in the SM of CC. GDF-15 (a member of the GDNF (Glial Cell-Derived Neurotrophic Factor) family) is produced by muscle cells and released into blood and it has been reported to be significantly elevated in cancer patients and to promote muscle loss [124,140]. Strategies based on antagonizing MSTN and activin A signaling have been developed using antibodies against ActRIIB and MSTN, namely bimagrumab and LY2495655, respectively, the latter of which is recommended for the treatment of CC, especially for muscle atrophy management [33,118,130,141,142]. Bimagrumab is a completely human monoclonal antibody, which was discovered to be widely employed against ActRIIB in the treatment of CC or muscle wasting disorders [143,144]. It was reported that a single intravenous dosage of bimagrumab (30 mg/kg) increased the recovery of thigh muscle volume [145]. LY2495655 is a humanized monoclonal antibody that inhibits myostatin and was developed to treat muscle wasting diseases. In older individuals with muscular weakness, LY2495655 therapy was demonstrated to enhance lean body mass and somewhat improve muscle performance [146,147].

### 4.11. Metabolism Modulators

Cachexia can be caused by accelerated protein degradation or suppressed protein synthesis and myogenesis in SM [120,137]. Three pathways have been suggested to be responsible for protein degradation at the cellular level in SM, namely, ubiquitin-mediated proteasome degradation, autophagy, and calcium-dependent calpain-mediated degradation [96]. In animal models of cachexia, IGF-1 levels were decreased, and cachexia was associated with the development of insulin resistance [16,96]. Targeting anabolic pathways is considered to provide a means of overcoming and reversing muscle loss by modulating IGF-1 signaling and regulating ubiquitin ligase (E3). Trials have been initiated on the appetite-stimulating effects of medroxyprogesterone acetate and megestrol acetate [85,130].

### 4.12. Non-Pharmacological Treatment Option

Aerobic exercise is considered to improve insulin sensitivity and glucose homeostasis and has been suggested as a therapy for CC [141,142,148]. The effects of treadmill exercise on CC have been investigated in animals [149,150]. Jee et al. reported that intense exercise improved quality of life, survival rate, and muscle atrophy in mice [149], and Moreira et al. showed that regular treadmill running for 8 weeks reduced tumor growth and cachexia and improved insulin sensitivity in Walker 256 tumor-bearing adult rats [150].

## 5. Concluding Remarks

Cancer cachexia (CC) has many possible causes, and its presence should be detected early to maximize the effectiveness of treatments. The treatment of CC is multimodal and includes physical exercise, MSTN blockade, medications, and nutritional supplements, because single approaches are invariably ineffective. Strategies based on the regulation of IGF-1 and the suppression of MSTN increase muscle growth and aid recovery from cachexia, and thus, drugs and inhibitors used to treat cachexia should have anti-inflammatory and appetite-stimulating properties. In summary, full understanding of the molecular mechanisms, signaling pathways, and the secondary causes of muscle and/or fat wasting are essential for successful CC management.

## Figures and Tables

**Table 1:** List of therapeutic agents and factors available for the management of cancer cachexia.

Treatment Options	Level Decrease	Level Increase	References
Omega-3 fatty acids	decrease TNF-α and IL-1	recover the ability of nutrition	[91,92,93,94,95]
Glucocorticoids	prevent the synthesis/discharge of proinflammatory cytokines		
Non-steroidal anti-inflammatory drugs	reduce inflammation	reduce muscle wasting	
Drugs (cytokine inhibition)			
Glutamine supplementation		can reduce muscle wasting in cancer patients	[96,97,98]
Megestrol, Dronabinol		increase weight	[99]
Appetite stimulation (cannabinoids or erythropoietin)		ameliorate cachexia	[93,94,100]
Anti-dopaminergics (like metoclopramide			
Muscle creation stimulation (branched-chain amino acids			
Exercise (strength and aerobic training)	reduces proinflammatory cytokine levels	increases anti-inflammatory cytokine levels	[101]
Ghrelin agonists		therapeutic targeted approaches that reduce wasting in cancer patients	[21]
Androgen receptor agonists			
β-blockers			
anti-MSTN peptides			
Ghrelin analogs	reduce systemic inflammation and muscle catabolism	increase food intake and aid lean body mass retention	[102]
MSTN blockade	reduces inflammation and muscle wasting		[102]
Blockade of Stat3	reduces muscle atrophy and inflammatory cytokine expression		[100]
